# Fecal Bacterial Community Changes Associated with Isoflavone Metabolites in Postmenopausal Women after Soy Bar Consumption

**DOI:** 10.1371/journal.pone.0108924

**Published:** 2014-10-01

**Authors:** Cindy H. Nakatsu, Arthur Armstrong, Andrea P. Clavijo, Berdine R. Martin, Stephen Barnes, Connie M. Weaver

**Affiliations:** 1 Department of Agronomy, Purdue University, West Lafayette, Indiana, United States of America; 2 Department of Nutrition Science, Purdue University, West Lafayette, Indiana, United States of America; 3 Department of Pharmacology and Toxicology, University of Alabama at Birmingham, Birmingham, Alabama, United States of America; The Chinese University of Hong Kong, Hong Kong

## Abstract

Soy isoflavones and their metabolism by intestinal microbiota have gained attention because of potential health benefits, such as the alleviation of estrogen/hormone-related conditions in postmenopausal women, associated with some of these compounds. However, overall changes in gut bacterial community structure and composition in response to addition of soy isoflavones to diets and their association with excreted isoflavone metabolites in postmenopausal women has not been studied. The aim of this study was to determine fecal bacterial community changes in 17 postmenopausal women after a week of diet supplementation with soy bars containing isoflavones, and to determine correlations between microbial community changes and excreted isoflavone metabolites. Using DGGE profiles of PCR amplified 16S rRNA genes (V3 region) to compare microbial communities in fecal samples collected one week before and one week during soy supplementation revealed significant differences (ANOSIM p<0.03) before and after soy supplementation in all subjects. However, between subjects comparisons showed high inter-individual variation that resulted in clustering of profiles by subjects. Urinary excretion of isoflavone (daidzein) metabolites indicated four subjects were equol producers and all subjects produced O-desmethylangolensin (ODMA). Comparison of relative proportions of 16S rRNA genes from 454 pyrosequencing of the last fecal samples of each treatment session revealed significant increases in average proportions of *Bifidobacterium* after soy consumption, and *Bifidobacterium* and *Eubacterium* were significantly greater in equol vs non-S-(-)equol producers. This is the first *in vivo* study using pyrosequencing to characterize significant differences in fecal community structure and composition in postmenopausal women after a week of soy diet-supplementation, and relate these changes to differences in soy isoflavones and isoflavone metabolites.

**Trial Registration:**

Clinicaltrials.gov NCT00244907

## Introduction

Dietary consumption of soy isoflavones has been broadly studied for their potential health benefits [1], [2], [3], [4] mainly because of their similarity in chemical structure to human estrogens and affinity for estrogen receptors [5], [6]. In plants they are mainly found as two glycoside conjugates, 7-ß-D-glucosides and 6″-O-malonyl-7-ß-D-glucosides (i.e., daidzin, genistin and glycitin) and very little de-glycosylated forms (daidzein, genistein and glycitein). Fermentation by intestinal microbiota is required to produce the more biologically active forms, S-(-)equol and O-desmethylangolensin (ODMA) [6]. S-(-)Equol has been studied the most because it is thought to have the highest estrogenic activity, but only 30–60% of individuals have been reported to harbor the necessary microbiota for this bioconversion [7] whereas 80–95% of individuals are thought to be ODMA producers [8]. Little research has shown direct involvement of ODMA with any disease, but it has been hypothesized that the microbiota involved in metabolism of daidzein to ODMA may be important in the metabolism of other substrates that can contribute to health [8].

Only a couple of studies have examined the effect of soy intervention on gut microbiota in postmenopausal women [9], [10]. A short-term (5-d) soy treatment assessed using qPCR found a significant positive correlation between strong equol production and sulphate reducing bacteria and a negative correlation with the *Clostridium coccoides-Eubacterium rectale* group [9]. Whereas using a FISH approach for microbial analysis, significant increases in *Bifidobacterium*, the *Lactobacillus-Enterococcus* group and *Faecalibacterium prausnitzii* occurred after a month of soy consumption [10]. The length of soy treatment, groups targeted for assessment and method used differed in these two studies and are factors likely contributing to the different findings.

Most gut microbiota capable of metabolizing soy products or soy isoflavones to metabolites have been found using *in vitro* studies and *in vivo* animal studies [7]. Single bacterial isolates capable of metabolizing daidzein to S-(-)equol [11], [12] and daidzein to ODMA [13] have been identified using traditional fecal laboratory enrichments. On the other hand, others have found that a mixture of bacteria is needed to convert daidzein to S-(-)equol [14]. Using a human gut simulator, this mixture of bacteria (*Enterococcus faecium*, *Finegoldia magna*, *Lactobacillus mucosae*, and *Veillonella* sp.) has been used to convert daidzein to S-(-)equol when mixed with feces of non-S-(-)equol producers [15]. Based on these findings it is unclear if single or mixtures of bacteria more commonly carry out isoflavone metabolism in humans.

To better understand differences in gut microbiota *in vivo* response to soy consumption and bacterial populations contributing to variability in isoflavone metabolism, a more comprehensive approach is needed. The main aim of this study was to determine the effect of diet supplementation with soy on the fecal microbial community in postmenopausal women, and to determine the correlation between fecal bacterial populations with excreted soy isoflavone metabolites. A two-week experiment was conducted in which subjects in the first week were monitored for normal daily changes in fecal microbial community structure and the second week changes associated with the daily addition of soy bars to the diet using DGGE of 16S rRNA gene sequences (V3 region) amplified by PCR. Pyrosequences of the 16S rRNA gene (V3–V4 region) was used to compare bacterial community composition before and after diet supplementation with soy and to identify populations associated with metabolites produced after fermentation of isoflavones in postmenopausal women. Instead of targeting specific populations, which is a more limited approach, we chose to evaluate more comprehensively all populations that change with soy consumption to identify those that are associated with isoflavone metabolite production.

## Materials and Methods

### Subjects

The protocol for this study and supporting checklist are available as supplemental materials; see Protocol S1 and Checklist S1. Subjects for this research were enrolled in a larger study conducted at Purdue University designed to determine the effect of soy isoflavones on bone calcium balance (NCT00244907, clinicaltrials.gov). The data for this protocol were collected from the participants before randomization for the larger trial [16]. Subjects were initially recruited, from January 2006 to September 2008, in response to flyers and local newspaper advertisements. General good health was verified by standard blood and urine chemistries. Exclusion criteria included <4 years post-menopause, allergy or sensitivity to soy products, and having taken antibiotics within three months previous to the study. Other exclusionary criteria related to the primary study included broken bones within the last 6 months, abnormal results from a mammogram within the last 12 months, and taking medication known to interfere with calcium metabolism. Subjects were asked to exclude soy from their diets for one week prior to beginning the trial. All women signed a written consent that was approved by the Institutional Review Board of Purdue University. A subset of 17 white women, age 60.2±7.3 y (mean ± standard deviation) and 13.5±7.9 y post menopause provided fecal samples for this study. Average height and weight were 165.9±6.3 cm. 74.2±16.7 kg respectively with a BMI of 26.9±6.4 (4 of the 17 subjects had BMI >30).

### Experimental design

The study period was comprised of two one-week sessions in which subjects were asked to avoid soy product consumption in their regular diet. During the first week individuals consumed their regular diets (designated no-soy samples) excluding soy products from their diet and in the second week they supplemented their regular diet daily with one Revival soy bar (160 mg of soy isoflavones and 1 g saponin) (designated soy samples). The timeframe used for the soy intervention is supported by work of others that have shown changes in gut microbial communities after a day of fat/fiber dietary interventions [17].

Urine samples were collected and processed as described previously for isoflavone metabolite analysis [18]. Samples were collected on day four of diet supplementation with soy bars and analyzed after hydrolysis with *H. pomatia* B-glucuronidase/sulfatase for the metabolites genistein, daidzein, dehydrodaidzein, glycitein, formononetin, biochanin A, coumestrol, O-desmethylangolensin (O-DMA), 6-hydroxy-ODMA and S-(-)equol. Metabolites were measured using reverse-phase HPLC in tandem with electrospray ionization mass spectrometry using a AB Sciex 4000 triple quadrupole mass spectrometer (AB Sciex, Concord, Canada) as described previously [19]. Subjects were considered to be S-(-)equol producers if the Log10 transformed S-(-)equol:daidzein ratio (Log10 E:D) was higher than −1.75 according to Setchell [20]. Full data sets of metabolites excreted by three subjects (331, 341, and 355) were not available and therefore were not included in metabolite comparisons or in correlations with bacterial community composition.

### Fecal processing

During the study fecal samples were collected daily by subjects and kept on ice, for no more than 12 hours, until taken to Purdue University and stored at −20°C until processed. Each individual provided at least 13 samples during the course of the study. Fecal samples were thawed at 4°C overnight before processing. Each fecal sample was weighed, placed in a sterile stomacher bag and homogenized for approximately 2 minutes with two volumes of sterilized Milli-Q water per weight using a stomacher apparatus. Samples were aliquoted into sterile polypropylene tubes from the DNA extraction kit and stored at −20°C until DNA extraction.

### DNA extraction

Total genomic DNA was extracted from each fecal homogenate (100 µL) using the Fast DNA Soil Spin kit (Q-BIO 101, Carlsbad, CA) according to the manufacturer’s instructions. We have previously demonstrated that this kit yields maximum DNA from fecal samples due to the mechanical lysis process included as part of the protocol [21]. Quality of DNA was checked by electrophoresis using 0.8% agarose gels stained with 5 µg/mL ethidium bromide. DNA concentration was determined by fluorometry using a NanoDrop 3300 and the 260/280 OD ratio was calculated by spectrometry using a NanoDrop 1000 (Thermo Scientific, Wilmington, DE).

### PCR Denaturing Gradient Gel Electrophoresis, PCR-DGGE

Fecal community structure was determined using PCR-DGGE. A ∼181 bp fragment from the V3 region of the 16S rRNA gene was amplified by PCR using the universal bacterial primers PRBA338F-GC and PRUN518R [22], [23] (Table S1). The PCR solution included 1 ng of purified DNA, 0.37 picomoles of each primer, 1.25 units of Taq DNA Polymerase (NEB, Ipswich, MA), 1.5 mM MgCl, 0.8 mM of dNTP’s (Promega, Madison, WI) and 0.1% of BSA (Amresco, Solon, OH) in a final volume of 50 µL. The cycling parameters were comprised of an initial denaturation at 94°C for 5 minutes, followed by 30 cycles of denaturation at 92°C for 30 seconds, annealing at 55°C for 30 seconds and extension at 72°C for 30 seconds. Final extension was done at 72°C for 7 minutes. PCR products size, quality and quantity were checked by electrophoresis in 1.2% agarose gels stained with ethidium bromide. Equivalent amounts of PCR product were run in 8% acrylamide gels in the DCode Universal Mutation Detection System (Bio-Rad, Hercules, CA), using denaturing gradients of 30%–50%, 45%–55% and 45%–65% (7M of urea and 40% of formamide corresponded to 100% denaturant). Gels were run at 60°C for 5 hours at 200 volts and then stained in a solution of 1X of SYBR® Green I nucleic acid gel stain (Invitrogen, Eugene, OR). The gels were digitalized and analyzed using BioNumerics software with a 4% intensity cutoff for band detection (Applied Maths, Austin, TX). Similarities between PCR-DGGE profiles were calculated by absence or presence of bands, using the Dice coefficient and dendrograms were generated using the UPGMA algorithm.

### Pyrosequencing

The 454 FLX system (Roche, Branford, CT) was used to determine bacterial composition of the last fecal samples from each treatment session for each subject. The V4 region of the 16S rRNA gene was amplified by PCR using 10 picomoles of a 8-mer tagged forward primer 520-F and a set of four reverse primers 802-R [24], [25] (Table S1). PCR included 0.01 units/µL of Phusion HF polymerase (NEB, Ipswich, MA), 1X HF buffer, and 0.2 mM dNTPs (Promega, Madison, WI). The thermal cycles used were 95°C for 5 min followed by 30 cycles of 95°C for 30 sec, 60°C for 30 sec and 72°C for 1 min followed by 72°C for 5 min. The amplified product was purified after electrophoresis in agarose gels using the QIAEX II extraction kit (QIAGEN, Germantown, MD). Amplicon libraries from each sample were quantified, pooled at equimolar concentrations and sequenced using standard 454 FLX pyrosequencing at the Purdue Genomics Facility. Sequences were deposited in MG-RAST [26] under the accession ID numbers: 4579405.3, 4579406.3, 4579407.3, 4579408.3, 4579409.3, 4579410.3, 4579411.3, 4579412.3, 4579413.3, 4579414.3, 4579415.3, 4579416.3, 4579417.3, 4579418.3, 4579419.3, 4579420.3, 4579421.3, 4579422.3, 4579423.3, 4579424.3, 4579425.3, 4579426.3, 4579427.3, 4579428.3, 4579429.3, 4579430.3, 4579431.3, 4579432.3, 4579433.3, 4579434.3, 4579435.3, 4579436.3, 4579437.3, 4579438.3.

### Sequence Analysis

Sequences obtained from the 454 FLX pyrosequencing were analyzed using the QIIME pipeline (Version 1.7.0, http://qiime.sourceforge.net/) [27]. Sequences shorter than 150 bp, with any ambiguous bases or containing errors in the primer were filtered out. Furthermore, low quality sequences were identified by denoising [28] and filtered out of the dataset. OTUs were selected using the usearch61 clustering method, that also identifies chimera sequences, [29] by aligning with the Greengenes reference dataset (97% sequence similarity threshold) using the python implementation of the NAST algorithm, PyNAST [27]. Taxonomic classification of representative sequences from each OTU was carried out using the RDP Bayesian classifier [30] with 50% confidence and Greengenes taxonomy [31] version 13_5. Representative sequences were used to construct a phylogenetic tree through fast-tree. Equivalent numbers of sequences were obtained by rarefaction (10 iterations) to the lowest number of sequence reads found among the sample datasets and were used for all subsequent analyses. Good’s coverage was used to ensure that the majority of taxa in each subject were included in the rarefied dataset comparisons. Alpha diversity comparisons were performed using species richness estimators Chao1 and observed species, the Shannon diversity index, and the PD whole tree phylogenetic metric. For beta diversity comparisons rarefied sequence datasets were used to calculate Unifrac phylogenetic distances (Unifrac G-full-tree, weighted, weighted-normalized, unweighted, unweighted full-tree) [32] and non-phylogenetic distances (Bray Curtis and Euclidean).

### Data analysis

Analysis of similarity (ANOSIM) [33] was used to compare Dice coefficients from PCR-DGGE profiles to determine if the fecal bacterial community structures were significantly different (p<0.05) before and after supplementation with soy. For each subject the last four samples from the no-soy treatment were compared to the last four samples from the soy treatment. The last four samples were used to represent the changed microbial community structure after soy consumption. The Paleontological Statistics package version 2.16 (PAST) was used for these purposes [34]. T-test was used to determine significant differences in the number of bands obtained from PCR-DGGE profiles within and between subjects.

Wilcoxon signed-rank test was used to determine differences in the relative proportions of bacterial population obtained by pyrosequencing before and after soy diet supplementation. Mann-Whitney was used to determine differences in relative abundances of bacterial populations between S-(-)equol and non-S-(-)equol producers after supplementation with soy and differences in isoflavone metabolites that were excreted from S-(-)equol and non-S-(-)equol producers. Kendall tau rank correlation coefficients were calculated to identify significant correlations (p<0.05) between metabolites excreted and fecal bacterial populations determined by pyrosequencing. All these basic statistical tests were performed using software available in PAST [34]. Statistically significant differences between beta diversity distances were performed using PERMANOVA [35] and PERMDISP (permutational analysis of multivariate dispersions) [36] to ensure differences were not due to dispersion; both programs are available in the QIIME pipeline. Canonical correspondence analysis (CCA) [37] in the PC-ORD software package (Gleneden Beach, OR) was used to determine associations between the metabolites excreted and proportional average abundances of bacterial populations in each soy sample. Subjects 331, 341 and 355 were excluded from CCA and correlations calculations because complete metabolite excretion datasets were not available. Significant differences for the correlations were calculated using a Monte Carlo test with 999 iterations.

## Results

### Isoflavone metabolites excreted by postmenopausal women

Metabolites from isoflavones were detected from urine samples in postmenopausal women after 4 days of supplementation with soy (Table 1). The aglycones with the highest recovery in urine were daidzein (averaging 5223.3±2887.6 nM) and glycitein (averaging 3394.2±2077.9 nM). Concentrations of excreted S-(-)equol were highly variable between subjects ranging from 4.72 to 4155 nM. Log_10_ S-(-)equol:daidzein ratio of subjects 321, 325, 328 and 346 were greater than −1.75 making them S-(-)equol producers as defined by Setchell [20]. These subjects excreted significantly higher average amounts of S-(-)equol (2915 nM ±1558.6) than subjects considered being non-S-(-)equol producers (18.3 nM ±12.8) (P-value<0.05). The amounts of ODMA (O-desmethylangolensin) excreted were also highly variable ranging from 22.4 to 3375 nM (average 1503.8±1081.3). There is currently no cutoff established for ODMA producers, but using the ratio (OMDA:daidzein >0.018) used by Guo [38] all subjects would be considered to be OMDA producers. S-(-)Equol producers excreted significantly (P-value <0.05) lower amounts of ODMA and dihydrodaidzein compared to non-S-(-)equol producers. Detectable concentrations of coumestrol were not found. No significant differences were found for age, height, weight and BMI averages between S-(-)equol and non-S-(-)equol producers.

**Table 1:** Soy isoflavone metabolite concentrations (nM) in urine on day four of soy bar consumption.

Subject ID	Equol	Daidzein	DHDaid	ODMA	Genistein	Glycitein	Formononetin	Biochanin-A	6OH-ODMA
128	14.5	4907.5	1310.0	1531.5	227.0	2345.0	0.4	nd	114.0
301	52.6	5950.0	1570.0	1035.0	915.0	2772.5	0.5	0.4	269.0
303	10.8	4472.5	1415.0	220.3	352.8	1422.5	0.3	0.3	52.0
310	22.3	10650.0	1495.0	2340.0	2150.0	6100.0	1.4	1.7	140.5
321	4342.5	4397.5	570.0	800.0	690.0	5875.0	0.4	1.0	191.0
325	1302.5	213.8	198.5	22.5	67.8	278.8	0.2	0.4	70.5
328	1860.0	8100.0	1302.5	1237.5	812.5	3772.5	0.7	0.7	373.3
330	21.1	1972.5	1062.5	2790.0	159.5	426.8	0.7	0.6	101.3
335	4.7	3680.0	1327.5	2785.0	219.5	2290.0	1.8	0.3	72.3
338	14.9	7975.0	1382.5	2007.5	2165.0	4645.0	0.5	0.6	102.3
339	23.2	5675.0	1772.5	2082.5	892.5	3222.5	0.6	0.5	150.5
342	9.1	3077.5	1317.5	647.5	246.3	2892.5	0.3	0.6	49.0
344	18.9	3105.0	1250.0	3375.0	537.5	4225.0	0.2	nd	257.5
346	4155.0	8950.0	765.0	179.8	nd	7250.0	0.9	1.5	79.8

Metabolites concentrations were determined using reverse-phase HPLC in tandem with electrospray ionization mass spectrometry.

DHDaid = Dihydrodaidzein, ODMA = *O*-desmethylangolensin, 6OH-ODMA = 6-hydroxy-O-desmethylangolensin.

nd = below detection limit.

Full datasets for subjects 331, 341, and 355 were not available.

Coumestrol concentrations were below the detection level.

Pearson’s correlations were found to be significantly different between concentrations of some isoflavones and isoflavone metabolites (Table S2). Positive correlations were found between daidzein and genistein (0.66, p = 0.01), glycitein (0.76, p = 0.002) and biochanin A (0.67, p = 0.009). A negative correlation was found between S-(-)equol and dihydrodaidzein (−0.66, p = 0.01).

### Community structure comparisons using 16S rRNA gene PCR-DGGE

**Figure 1:**
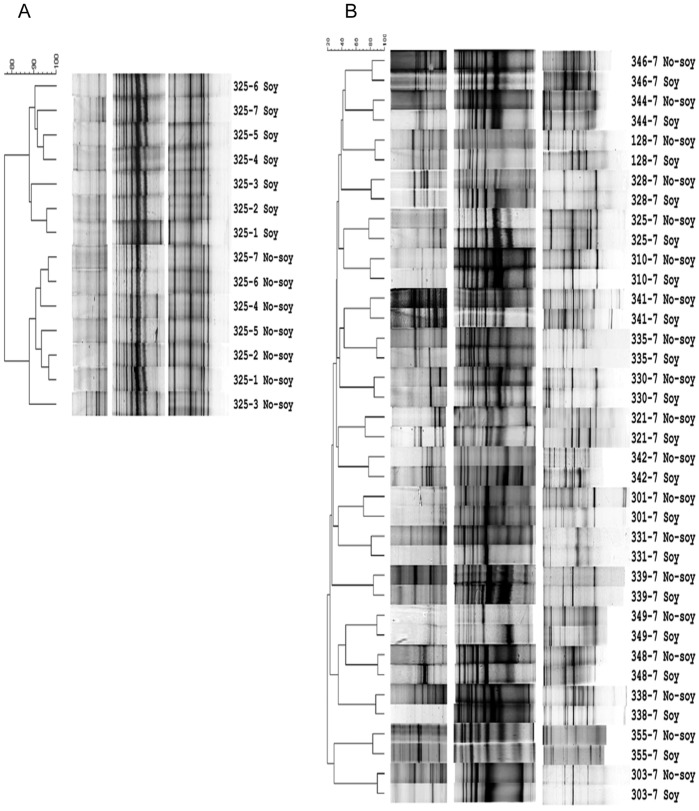
UPGMA dendrograms of 16S rRNA gene PCR-DGGE profiles of fecal samples collected from postmenopausal women. (A) Representative examples of within subject comparisons (subject 325). (B) Comparison between all subjects using the last samples from each treatment session. Each profile is labeled by subject number, collection day and soy treatment. Dendrograms were constructed using UPGMA of pairwise Dice similarities comparisons between samples. ‘No-soy’ samples represent the samples before the supplementation with soy and, ‘Soy’ samples refers to samples collected during the week of supplementation with soy.

The range of Dice similarity coefficients and the number of bands in PCR-DGGE profiles during the no-soy and soy weeks indicated there was high variability between subjects (Table 2). The UPGMA comparison of Dice coefficients between no-soy and soy diet samples from the postmenopausal women illustrated clustering of PCR-DGGE profiles by soy diet-supplementation (example, Figure 1A). Change in community structure from the soy diet varied among subjects; distinct changes in profiles were seen 1 to 4 on days after starting the soy intervention. ANOSIM analysis of Dice coefficients of PCR-DGGE profiles between no-soy and soy samples showed that within subjects there were significant (*P*<0.03) differences in bacterial community structure in all the subjects (Table 2). The UPGMA comparison of the end PCR-DGGE profiles for soy and no-soy treatments clearly illustrated clustering of profiles first by subject and then by soy treatment (Figure 1B). This was the same clustering found when all PCR-DGGE profiles from all subjects were compared (data not shown).

**Table 2:** Comparison of 16S rRNA gene PCR-DGGE profiles between no-soy and soy samples for each subject.

	Range of # of bands		ANOSIM β	
Subjects	No-soy	Soy	R value	P- values
128	40–36	43–32	0.7500	0.0280
301	40–31	38–34	1.0000	0.0280
303	34–31	34–28	0.8750	0.0280
310	39–36	33–28	1.0000	0.0284
321	47–37	43–33	0.8542	0.0285
325	32–28	36–32	1.0000	0.0300
328	44–33	43–34	0.9896	0.0290
330	31–28	43–34	0.8958	0.0300
331	30–27	27–22	0.9323	0.0300
335	43–39	43–40	1.0000	0.0290
338	48–42	47–44	0.6302	0.0290
339	32–27	39–33	0.4115	0.0290
341	66–59	72–59	1.0000	0.0290
342	45–38	39–33	0.9375	0.0290
344	39–34	44–37	0.8542	0.0290
346	52–45	52–45	0.3958	0.0280
355	33–26	35–31	0.5365	0.0290
Mean	37.35	37.94	0.8772	0.0289

β Significant differences (P<0.05) determined using ANOSIM comparison of Dice coefficients from last four samples for each subject to capture the changed microbial community structure after addition of dietary soy bars.

R statistic can range from −1 to +1 with 1 indicating that the most similar values are within each group being compared.

### Fecal bacterial community composition and differences in populations after soy consumption

Pyrosequencing of the V4 region of the 16S rRNA gene of fecal bacterial community after denoising and chimera check yielded a total of 325,636 sequences with an average of 9,618 (±2,908) sequences per sample and ranged from 4,900 to 16,250 sequences. All calculations and community comparisons were made using a subset of 4,900 sequences for each sample that were selected randomly after 10 iterations of rarefaction. More than 99% of the sequences were classified into the phyla Actinobacteria, Bacteroidetes, Firmicutes, Proteobacteria and Verrucomicrobia, for both no-soy and soy samples. Other phyla present in percentages less than 0.001% included Cyanobacteria, Synergistetes, Tenericutes and TM7. Although primers used were theoretically specific for Bacteria some Archaea sequences were also amplified but were not included in downstream analysis due unknown coverage of this phylum by these primers.

Taxonomic assignment of the sequences in all samples resulted in the identification of 65 genera, and an additional 47 taxa that were not classified to a known genus (designated “other” or “unclassified”) (Table S3). Comparing all subjects together the average relative proportions of three genera significantly changed after a week of soy intake; *Bifidobacterium* increased and *Lactobacillus* and unclassified Clostridiaceae decreased (Table 3). A comparison of subjects grouped into S-(-)equol and non-S-(-)equol producers also showed significant differences in relative abundances of six taxa at the end of a week of soy intake (Table 4), in this comparison *Bifidobacterium, Rothia,* other Bifidobacteriaceae, other Actinobacteria, and unclassified Streptophyta were significantly higher (P<0.05) in S-(-)equol producers whereas *Roseburia* was significantly lower.

**Table 3:** Bacterial genera that significantly differed in relative proportions after soy consumption by postmenopausal women.

	Treatment	
Genus	No-soyMean (SD) %	SoyMean (SD) %
*Bifidobacterium*	4.45 (5.13)	7.53 (9.49)
Unclassified Clostridiaceae	1.53 (2.58)	0.14 (0.22)
*Lactobacillus*	0.15 (0.19)	0.06 (0.11)

Significant difference at P<0.05 determined using Wilcoxon signed-rank test.

**Table 4:** Bacterial genera that significantly differed in relative proportions after soy consumption by S-(-)equol versus non-S-(-)equol producers.

*Genus*	Non-S-(-)equol producers Average (SD) %n = 13	S-(-)Equol producersAverage (SD) %n = 4
*Bifidobacterium*	4.08 (6.27)	18.73 (13.54)
*Rothia*	0.001 (1.72)	0.012 (3.24)
*Roseburia*	1.80 (2.91)	0.23 (0.24)
Other Bifidobacteriaceae	0.01 (0.00)	0.05 (0.008)
Other Actinobacteria	0.01 (0.00)	0.02 (0.04)
Unclassified Streptophyta	0.0005 (0.18)	0.01 (0.17)

Significant difference at P<0.05 determined using Mann Whitney unpaired test.

Using rarefaction the same number of sequences from each sample was used in comparisons of community alpha and beta diversity measures. An average of 98.7% (range 97.7–99.4%) Good’s coverage was calculated for the rarefied dataset. Paired t-tests indicated there were no significant differences between subjects on the no-soy and soy diets based on the alpha diversity measures: Shannon diversity index (4.9±0.6 vs 4.6±0.9), richness indices ChaoI (432.8±110.8 vs 440.3±112.7) and observed species (305.4±79.0 vs 302.9±81.4), and phylogenetic diversity (PD) whole-tree measure (20.6±4.2 vs 20.8±4.8) between no-soy vs soy samples, respectively.

**Figure 2:**
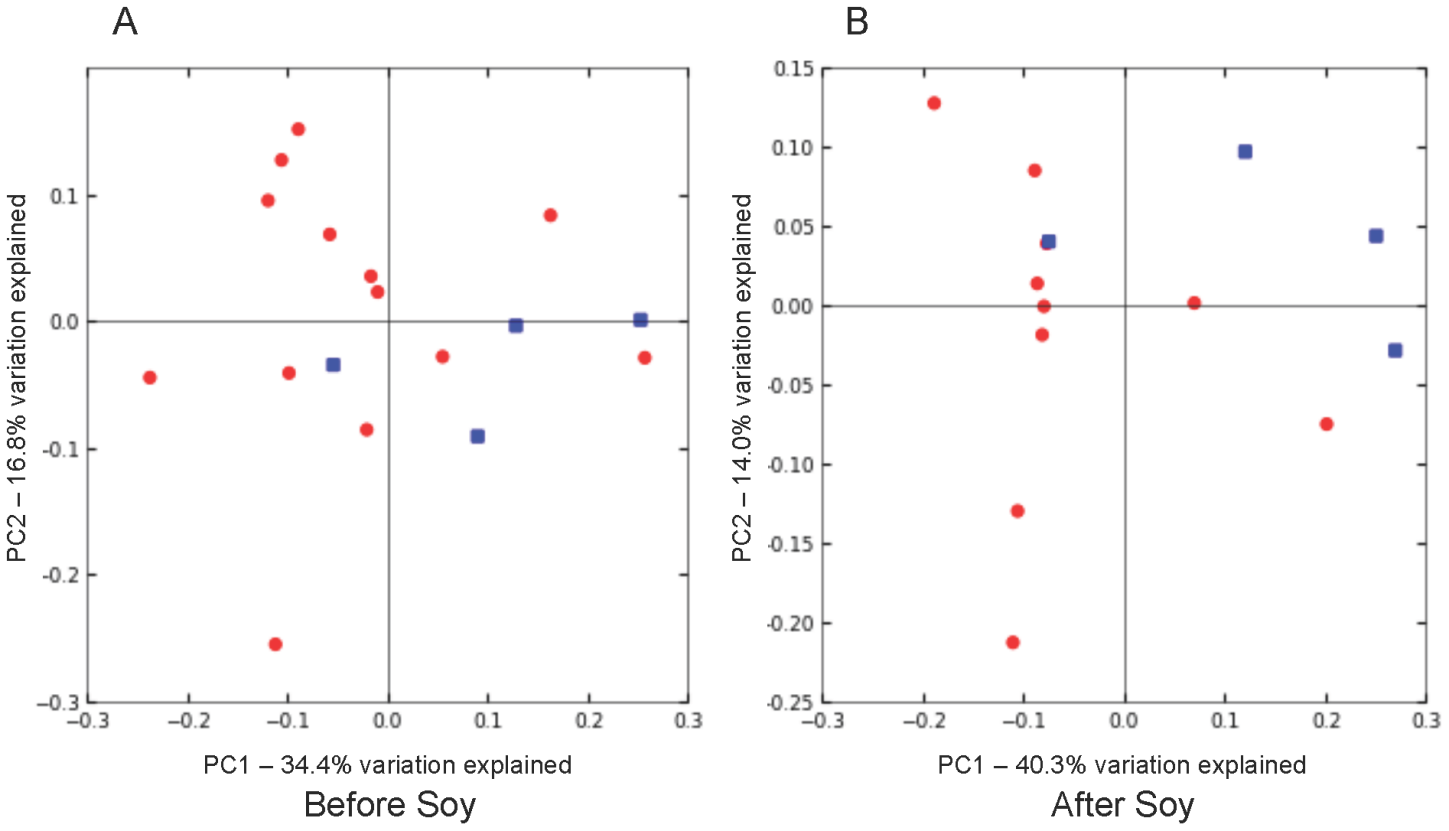
Principal coordinate analysis (PCoA) of weighted Unifrac distances of subjects (A) before soy consumption and (B) after soy consumption. Non-S-(-)equol producers are represented by red dots, and blue dots represent S-(-)equol producers.

Principal coordinate analysis (PCoA) of weighted Unifrac distances illustrated that communities of S-(-)equol producers clustered more together at the end of the soy diet that was not as evident before the soy diet when they were more intermixed (Figure 2A and 2B). Results from PERMANOVA indicated differences in weighted Unifrac distances were significant (p<0.05) and further testing using PERMDISP indicated that dispersion was not contributing significantly to these differences. However, the differences between S-(-)equol and non-S-(-) equol producers were not significant using Bray Curtis or unweighted Unifrac distances. Additionally, beta diversity comparisons did not show clear clustering between communities at the ends of the soy versus no-soy treatments (data not shown).

### Correlations between bacterial communities and excreted isoflavone metabolites after soy consumption

Average relative proportions of 30 taxa significantly correlated with one to three excreted isoflavone metabolites (Table S4). Seventeen taxa correlated positively and 14 negatively with at least one metabolite. In addition to previously unreported taxa involved in isoflavone metabolism (designated by ψ in Table 5), there were correlations of bacterial genera with daidzein and its metabolites dihydrodaidzein, S-(-)equol and OMDA that showed some potential relationships between metabolites. The group designated “other” Bifidobacteriaceae was negatively correlated with dihydrodaidzein and positively correlated with S-(-)equol, whereas, *Peptoniphilus* was negatively correlated with S-(-)equol and positively correlated with ODMA. Also notable was *Bifidobacterium*; it correlated negatively with dihydrodaidzein (−0.56) and was found to significantly increase in subjects after soy supplementation (Table 2).

**Table 5:** Bacteria taxa with relative proportions correlating significantly with concentration of daidzein and daidzein metabolites.

Phylum	Family	Genus	Daidzein	DHdaidzein	S-(-)Equol	ODMA
Actinobacteria	Bifidobacteriaceae	*Bifidobacterium*		−0.559	0.179*	
		Other		−0.410	0.438	
	Coriobacteriaceae	*Adlercreutzia*			−0.451	
		*Collinsella*			0.536ψ	
		*Slackia*	−0.464			
	Corynebacteriaceae	*Corynebacterium*		−0.409		
	Micrococcaceae	*Rothia*			0.410ψ	
	Other	Other				−0.401
Firmicutes	Clostridiaceae	*Clostridium*	−0.431			
	Eubacteriaceae	*Pseudoramibacter*		0.398ψ		
	Erysipelotrichaceae	Other				0.451
	Lachnospiraceae	*[Ruminococcus]*			−0.407	
		*Dorea*				0.407ψ
	Peptostreptococcaceae	Unclassified	−0.438	−0.466		
	Ruminococcaceae	*Oscillospira*				0.473ψ
		Other				0.407
		*Ruminococcus*				0.451
	Streptococcaceae	*Lactococcus*			0.402	
	[Tissierellaceae]	*Finegoldia*				0.398ψ
		*Peptoniphilus*			−0.398ψ	0.466ψ
	Veillonellaceae	*Dialister*	0.524ψ			
	Other	Other			0.401	
Verrucomicrobia	Other	Other	−0.440			−0.398

Correlations calculated using the non-parametric Kendall’s tau measure and significant if p<0.05.

Full table of correlations with all isoflavone metabolites are available in Table S4.

*Correlation between *Bifidobacterium* and equol is not significant, reported here to illustrate the negative relationship between dihydrodaidzein and S-(-)equol.

ψ Genera not previously reported to be involved in isoflavone metabolism [7].

Groups listed as unclassified or other cannot be assigned to a genus at this time and therefore isoflavone metabolism is unknown.

DHdaidzein = dihydrodaidzein, ODMA = *O*-desmethylangolensin.

**Figure 3:**
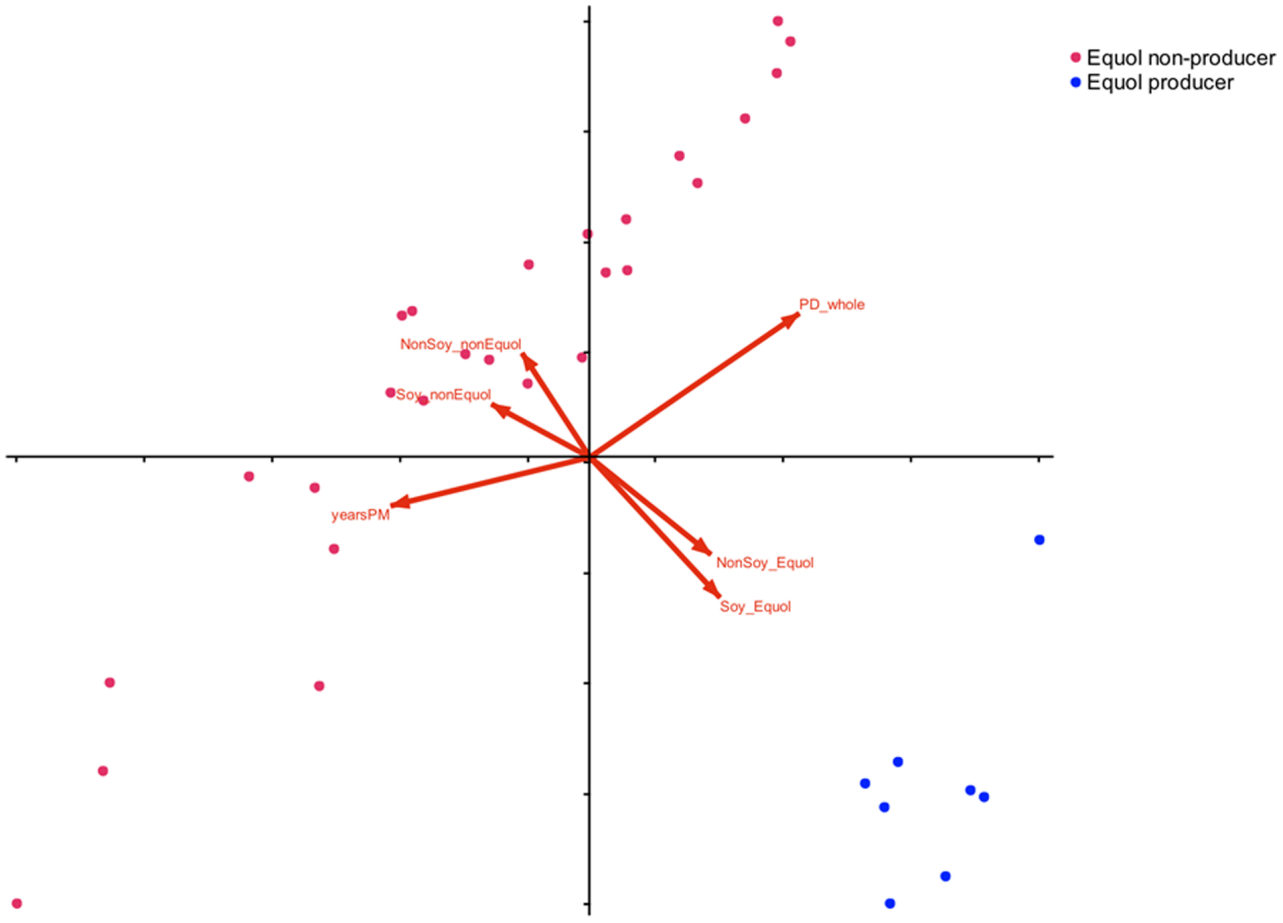
Canonical correspondence analysis of fecal bacterial composition and alpha diversity measures and postmenopausal women metrics. Non-S-(-)equol producers are represented by green dots, and red dots represent S-(-)equol producers. Arrows represent the direction of the host factors significantly corresponding to bacterial community composition. Host factors depicted are equol producers, non-S-(-)equol producers, with no soy in diet, after soy intervention, years post-menopause and alpha diversity (illustrated PD whole tree phylogenetic diversity but Chao1 and Shannon have the same trajectory). Variation explained in horizontal axis is 8.2% and the vertical axis is 6.9%.

Canonical correspondence analysis (CCA) was used to determine the relationship between the variation in bacterial community composition, and some subject traits that showed significant correlations. Host factors that significantly corresponded (p<0.05) with the microbial communities were being an S-(-)equol producer and the number of years the subject was post-menopause (Fig. 3). Arrows are in opposite orientations for S-(-)equol and non-S-(-)equol producers. In a perpendicular orientation are arrows for the number of years post-menopause that is opposite to alpha diversity measures (PD whole tree phylogenetic metric is given as an example but results are the same using Chao1 and Shannon measures).

## Discussion

After daily supplementation with a soy bar for one week the baseline fecal bacterial community structure and composition in postmenopausal women significantly changed. By using a community genetic fingerprinting method, PCR-DGGE, temporal changes were tracked and soy treatment differences were found to be significant. Based on the knowledge that community structure changed significantly, pyrosequencing of representative samples from the end of the soy and no-soy treatments were used to identify the bacterial genera contributing to these differences. There was a significant increase in the relative proportion of one genus, *Bifidobacterium*, after soy consumption. Furthermore, correlations were found between isoflavones and isoflavone metabolites with proportions of a number of different bacterial genera, many were previously not reported to be associated with isoflavone fermentation. The increase in proportion of *Bifidobacterium*, a bacterium found by others to be involved in soy fermentation, provides support for the validity of these new findings [10]. The negative correlation of *Bifidobacterium* with dihydrodaidzein suggests that it is involved in the fermentation of this metabolite to downstream metabolites. This study demonstrates that a week of a diet supplemented with soy is sufficient to significantly change the gut microbiome and these changes can be related to soy fermentation metabolites.

PCR-DGGE profiles indicated changes in bacterial community structure occurred from one to five days after beginning soy bar consumption, with the majority changing in the first couple of days. This illustrates subject variation in response to diet change but the effect is relatively rapid as seen in extreme changes in diet [39]. In addition, after supplementation with soy there was a reduction in dominant band numbers in PCR-DGGE profiles and reduction of diversity in community composition using pyrosequence data. These results suggested that soy was providing a selective environment leading to an increase in the proportion of a subset of the bacterial community. Pyrosequencing results analyzed using CCA indicated that number of years post-menopause was also contributing to a reduction in alpha diversity.

A potential bacterium that was selected by the soy diet was *Bifidobacterium* whose proportions increased with soy consumption, especially in S-(-)equol producers. Similar increases of *Bifidobacterium* in postmenopausal women after soy interventions have been previously reported [9], [10]. It also negatively correlated with dihydrodaidzein suggesting that in some of these subjects this bacterium was involved in the conversion of dihydrodaidzein to S-(-) equol. Another genus that is potentially involved in fermentation of isoflavones in postmenopausal women is *Rothia* that was in higher relative proportions in S-(-)equol versus non-S-(-)equol producers and was significantly correlated with equol production. This genus, also a member of the phylum Actinobacteria, has not been previously associated with S-(-)equol production. The proportions of this taxon are very low therefore more studies are needed in the future to determine its involvement in daidzein metabolism. Due to the high variability between subjects there is always a possibility that some of these correlations may be false positives.

Inherent variation between subjects in their gut microbiome and potential physiological redundancy of different bacterial taxa able to carry out isoflavone metabolism required additional analytical methods to explore associations between taxa and metabolites. Indeed, CCA indicated gut community composition corresponded with the number of years that subjects were post-menopause and was in the opposite orientation as alpha diversity measures, illustrating one potential source of gut microbiome variation among subjects. Correlation analyses identified a number of bacterial populations associated with isoflavone metabolism that have not been previously reported: *Collinsella, Dialister, Dorea, Finegoldia, Papillibacter. Peptoniphilus, Pseudoramibacter* and *Rothia*. Correlations also identified a number of genera that have been previously reported to metabolize isoflavones *in vitro*: *Adlercreutizia, Bifidobacterium, Clostridium, Corynebacterium*, *Lactococcus*, *Ruminococcus,* and *Slackia* (summarized in [7]). A human *in vivo* study targeting specific bacterial groups using FISH also showed the association between *Bifidobacterium* and S-(-)equol production in postmenopausal women [10]. But the *in vivo* metabolism of isoflavones and isoflavone metabolites in humans by these taxa has not been proven.

Lower relative abundances in the phylum Bacteroidetes were observed in the subjects in this study when compared with other studies that included subjects within the same age range (less than 0.04% in comparison with ∼10%) [40]. Fecal sample processing, including freezing of samples, has previously been considered to be a factor affecting recovery of Bacteroidetes [41]. However, fecal samples of adolescents from the same geographical region as the postmenopausal women under similar storage conditions provided higher number of Bacteroidetes [42]. Age could also be a contributing factor; other studies have also found variable results between elderly with younger age groups [40], [43]. Further analysis will be required to establish the influence of age to abundance of Bacteroidetes in postmenopausal women from the Midwest.

This is the first report using pyrosequencing to determine the *in vivo* response of GIT bacterial populations to one week of dietary soy supplementation in postmenopausal women. Previous studies examining the effect of soy on gut microbiota in postmenopausal women targeted only a few bacterial populations and monitored their changes. In this study using pyrosequencing positive correlations were found between bacterial taxa and production of soy isoflavone metabolites that have not been previously reported. Future studies in soy fermentation are needed to understand the specific role of these bacteria taxa. The validity of these findings was supported by increases in *Bifidobacterium* that have been reported in other studies. It negatively correlated with dihydrodaidzein and although not statistically significant its proportions correlated positively with excretion of S-(-)equol, suggesting a role in isoflavone metabolism in postmenopausal women. In addition, fecal bacterial communities from S-(-)equol and non-S-(-)equol producers were significantly different supporting the hypothesis that S-(-) equol production is dependent on intestinal bacterial community composition. Further studies are needed to determine the stability of changes in the gut microbiota in response to soy over longer periods of time and in the context different background diets, e.g., non-vegetarian versus vegetarian.

## Supporting Information

Table S1 **Primers used for fecal bacterial community analyses in postmenopausal women.**
(DOCX)

Table S2 **Correlations between isoflavones and isoflavone metabolite concentrations in urine samples from postmenopausal women after soy supplementation of diets.**
(DOCX)

Table S3 **Comparison of average proportion of genera in fecal samples of postmenopausal women after no-soy and soy supplementation of diets.**
(XLSX)

Table S4 **Correlations between average proportion of genera with isoflavones and isoflavone metabolites in fecal samples of postmenopausal women.**
(XLSX)

Checklist S1 **TREND Checklist.**
(PDF)

Protocol S1 **Trial Protocol.**
(PDF)
