# Supplementary material for: Fecal Bacterial Community Changes Associated with Isoflavone Metabolites in Postmenopausal Women after Soy Bar Consumption
Source: PLoS One. 2014 Oct 1;9(10):e108924. doi: 10.1371/journal.pone.0108924 (PMC4182758; doi:10.1371/journal.pone.0108924)
Supplement: Table S1 — Primers used for fecal bacterial community analyses in postmenopausal women. (DOCX) [file pone.0108924.s001.docx]

**Table S1. Primers used for fecal bacterial community analyses in postmenopausal women.**

| Method | Target | Primer | Sequence (5’ – 3’) | Product size (bp) | Reference |
| --- | --- | --- | --- | --- | --- |
| PCR-DGGE | Universal for Bacteria | PRBA338F | CCTACGGGAGGCAGCAG | 181 | (Muyzer et al, 1993) |
| PCR-DGGE | Universal | PRUN518R | ATTACCGCGGCTGCTGG | 181 | (Muyzer et al, 1993) |
| PCR-DGGE | Universal for Bacteria with GC clamp | PRBA338F-GC | CCTACGGGAGGCAGCAG CGCCCGCCGCGCGCGGCGGGCGGGGCGGGGGCACGGGGGG | 221 | (Muyzer et al, 1993) |
| Pyrosequencing | Universal for Bacteria | 520-F | AYTGGGYDTAAAGNG | 283 | (Claesson et al, 2009 ) |
| Pyrosequencing | Total Bacteria | 802-R | TACCRGGGTHTCTAATCC, TACCAGAGTATCTAATTC, CTACDSRGGTMTCTAATC, ACNVGGGTATCTAATC | 283 | (Cole et al, 2009) |

References

Muyzer G, de Waal EC, Uitterlinden AG (1993) Profiling of complex microbial populations by denaturing gradient gel electrophoresis analysis of polymerase chain reaction-amplified genes coding for 16S rRNA. Appl Environ Microbiol 59: 695-700.

Claesson MJ, O'Sullivan O, Wang Q, Nikkilä J, Marchesi JR, et al. (2009) Comparative analysis of pyrosequencing and a phylogenetic microarray for exploring microbial community structures in the human distal intestine. PLoS ONE 4: e6669.

Cole JR, Wang Q, Cardenas E, Fish J, Chai B, et al. (2009) The Ribosomal Database Project: improved alignments and new tools for rRNA analysis. Nucl Acids Res 37: D141-145.
