# Supplementary material for: Fecal Bacterial Community Changes Associated with Isoflavone Metabolites in Postmenopausal Women after Soy Bar Consumption
Source: PLoS One. 2014 Oct 1;9(10):e108924. doi: 10.1371/journal.pone.0108924 (PMC4182758; doi:10.1371/journal.pone.0108924)
Supplement: Table S2 — Correlations between isoflavones and isoflavone metabolite concentrations in urine samples from postmenopausal women after soy supplementation of diets. (DOCX) [file pone.0108924.s002.docx]

Table S2: Correlations between isoflavones and isoflavone metabolite concentrations determined from urine samples of postmenopausal women after soy supplementation of diets.

|  | Equol_ | Daidzein | Dihydrodaidzein | O_DMA | Genistein | Glycitein | Formononetin | Biochanin-A | 6_OH_O_DMA |
| --- | --- | --- | --- | --- | --- | --- | --- | --- | --- |
| Equol |  | 0.5259 | 0.0108 | 0.0680 | 0.4310 | 0.0379 | 0.9242 | 0.0630 | 0.6386 |
| Daidzein | 0.1853 |  | 0.1405 | 0.9456 | 0.0103 | 0.0017 | 0.1419 | 0.0089 | 0.3366 |
| Dihydrodaidzein | -0.6566 | 0.4146 |  | 0.1033 | 0.1184 | 0.9220 | 0.4613 | 0.5763 | 0.4928 |
| ODMA | -0.5011 | 0.0201 | 0.4536 |  | 0.2861 | 0.9998 | 0.1751 | 0.5234 | 0.4687 |
| Genistein | -0.2290 | 0.6592 | 0.4368 | 0.3068 |  | 0.1156 | 0.5181 | 0.1614 | 0.4128 |
| Glycitein | 0.5585 | 0.7576 | 0.0289 | 0.0001 | 0.4398 |  | 0.3983 | 0.0059 | 0.4225 |
| Formononetin | -0.0280 | 0.4133 | 0.2146 | 0.3841 | 0.1888 | 0.2451 |  | 0.1033 | 0.7311 |
| Biochanin-A | 0.5091 | 0.6691 | -0.1636 | -0.1864 | 0.3957 | 0.6936 | 0.4536 |  | 0.8793 |
| 6_OH_O_DMA | 0.1378 | 0.2776 | 0.2001 | 0.2111 | 0.2379 | 0.2331 | -0.1010 | -0.0447 |  |

Pearson's correlations are values below the diagonal and significance values are above the diagonal. Significance values <0.05 are highlighted in pink.

ODMA = O-desmethylangolensin

6_OH_O_DMA = 6-hydroxy-O-desmethylangolensin
