# Supplementary material for: Fecal Bacterial Community Changes Associated with Isoflavone Metabolites in Postmenopausal Women after Soy Bar Consumption
Source: PLoS One. 2014 Oct 1;9(10):e108924. doi: 10.1371/journal.pone.0108924 (PMC4182758; doi:10.1371/journal.pone.0108924)
Supplement: Protocol S1 — Trial Protocol. (PDF) [file pone.0108924.s006.pdf]

HS/3 - revised 4/97 Ref. # __________

Purdue University

Committee On The Use of Human Research Subjects

APPLICATION FOR APPROVAL TO USE HUMAN RESEARCH SUBJECTS

Please complete BOTH sides of this application form

1. Project Title: Effect of Soy Products on Calcium Metabolism in Humans

2. Full Review x Expedited Review ______ Procedural Revision ______

3. Anticipated Funding Agency: ______

4. Principal Investigator(s) *[Must be faculty member]*:

NAME and TITLE	Department, Building, Phone, FAX, E-mail address
Connie M. Weaver, Professor	F&N/Stone/weavercm@cfs.purdue.edu; 48231 ph/40674 fax
David Elmore, Professor	PHYS/PHYS/elmore@purdue.edu; 46516 ph/67228 fax
Munro Peacock, MD	IUPUI/mpeacock@iupui.edu; 317-274-4356 ph/317-274-7346 fax
Steve Badylak, MD	IAA/POTR/badylak@purdue.edu; 42995 ph

5. Other Personnel - such as consultants or graduate students *(add separate sheet if needed)*:

Name and Title	Department, Building, Phone, FAX, E-mail address
Berdine R. Martin, Laboratory Manager	F&N/Stone/martinb@cfs.purdue.edu/ 46559 ph/40674 fax
George Jackson, Ph.D.	~~Prime Lab/Physics/jackson1@physics.purdue.edu;~~

6. The principal investigator agrees to carry out the proposed project as stated in the application and to promptly report to the Human Subject's Committee any proposed changes and/or unanticipated problems involving risks to subjects or others participating in approved project in accordance with the Purdue Research Foundation-Purdue University Statement of Principles, and the Confidentiality Statement *(pages 4-5 of this document)*.

Connie Weaver
Principal Investigator Signature

2/17/00
Date

7. The Department Head (or authorized agent) has read and approved the application and agrees to maintain records for three years after completion of the project should the principal investigator terminate University association.

Connie M. Weaver
Department Head *(printed)*

Foods and Nutrition
Department Name

Connie Weaver
Department Head Signature

2/17/00
Date

Submit original and two copies of entire application to: Human Subjects Office, 1071 ENAD, Rm. 328.

8. This project will be conducted at the following SITE(S): (please indicate city & state)

- __x__ Purdue West Lafayette Campus
- _____ Purdue Regional Campus (Specify): __________
- _____ Hospital (Specify): __________
- _____ Other (Specify): __________

9. This project will involve the following subject types: (check-mark types to be studied)

- __x__ Normal Volunteers
- _____ In Patients
- _____ Out Patients
- _____ Patient Controls
- _____ Fetuses
- _____ Mentally Disabled
- _____ Physically Disabled
- _____ Pregnant Women
- _____ Subjects Incapable Of Giving Consent
- _____ Prisoners Or Institutionalized Individuals
- _____ Minors
- __x__ Over Age 65
- _____ Aborted Fetuses
- _____ Students (PSYC Dept. subject pool ____)
- _____ None Of The Above

10. This project involves the use of an **Investigational New Drug** (IND) or an **Approved Drug For An Unapproved Use.**

_____ YES __x__ NO

Drug name, IND number and company: __________

11. This project involves the use of an **Investigational Medical Device** or an **Approved Medical Device For An Unapproved Use.**

_____ YES __x__ NO

Device name, IDE number and company: __________

12. The project involves the use of **Radiation or Radioisotopes**:

__x__ YES _____ NO

13. Does this project call for: (check-mark all that apply to this study)

- __x__ Subject Compensation? Patients $_____ Volunteers $ 750/year
- _____ Advertising For Subjects?
- __?__ More Than Minimal Risk?
- _____ More Than Minimal Psychological Stress?
- _____ Alcohol Consumption?
- _____ VO2 Max Exercise?
- _____ Waiver of Informed Consent?
- __x__ Confidential Material (questionnaires, photos, etc.)?
- _____ Extra Costs To The Subjects (tests, hospitalization, etc.)?
- __x__ The Exclusion of Pregnant Women?
- __x__ The Use of Blood? Total Amount of Blood <50 mL every 50 days Over Time Period (days) _____

A) Brief Rationale

The specific objective is to determine the ability of soybean estrogens (phytochemicals which have estrogen-like activity) to increase calcium absorption and to enhance calcium retention in postmenopausal women. Osteoporosis is a debilitating disease affecting over 25 million Americans resulting in 1.3 million hip fractures annually. Annual health care costs related to hip fractures are approximately $13 billion. Current drugs on the market for treating osteoporosis have adverse side effects. If regular consumption of soybeans can improve calcium retention, this will reduce bone loss and lower the risk of osteoporosis. Such a finding could offer a choice besides taking estrogen or other drugs for postmenopausal women.

Because 99% of the body's calcium is in the bone, an understanding of calcium metabolism allows rather short term studies compared to bone density measurements which would require $\geq$ 1 year to observe changes due to an intervention.

B) Specific Procedures to Be Followed

Prior to the study, subjects will complete a general health questionnaire, diet food records, and a food frequency questionnaire. For each phase of the study, additional food records and food frequency questionnaires will be obtained and changes in physical activity and health/use of medications will be monitored. *Baseline blood samples will be drawn to measure general health through a chemical profile. Information will be reviewed by a physician prior to inclusion in the study. Bone density scans will be performed during the first year.*

Calcium and vitamin D supplements will be given throughout the study as needed to bring subjects up to recommended intakes. It is expected that this will typically involve 1 Viactive soft chew per day which contains 500 mg calcium as $CaCO_3$ and 100 IU vitamin D.

At least 2 months prior to beginning the baseline phase of the study, subjects will be given ^{41}Ca intravenously. Two months following administration of ^{41}Ca, the remaining isotope in the subject's body is considered to be part of the skeleton. Subsequently, ^{41}Ca appearing in the urine is a measure of bone resorption.

A baseline period will involve collection of up to 10 24-hour urine samples over 50 days. Each treatment phase will consist of asking each subject to consume a soy product daily for up to 50 days followed by a recovery period of up to 50 days. Subjects will be asked to collect up to five 24 hour urine samples during the treatment period and also during the recovery period.

Near the end of the baseline period and each treatment period (up to 4 times/year), subjects will be asked to participate in a calcium absorption test. This will involve consuming ^{44}Ca and giving a 5 mL blood sample at two time

points (time 0 and 5 hours). A 30 mL blood sample will be taken at the end of each treatment period and recovery period to measure biochemical markers of bone turnover, isoflavone concentrations, and isotopes.

The annual maximum participation for each subject would be:

	Baseline	Treatment	Recovery	Treatment	Recovery	Treatment	Recovery
DAY	50	100	150	200	250	300	350

The type of soy products to be tested are soy protein products enriched in isoflavones which are marketed by Protein Technologies International or companies which use their product under their own brand name. Soy isoflavone concentrates will also be tested. Manufacturers of these products include Protein Technologies International, Central Soya, and Archer Daniels Midland. Some companies such as Schouten, Inc. prepare soy isoflavone concentrates from soy germ which have a very different profile of isoflavones. The doses of isoflavones vary widely in the various products and manufacturers dosage suggestions are frequently given as ranges.

C) Type of Subjects to Be Employed

Healthy women at least 4 years postmenopausal will be recruited from the community. Exclusion criteria include women on hormone replacement therapy and those taking drugs known to alter calcium metabolism. Proposals will be written to study up to 20 subjects/year.

Subjects who fail to comply with the protocol will be terminated from the study. If a subject experiences a fracture during the study, they may not resume the study for one year. If a subject has a persistent adverse reaction to a soy product they will be terminated from the study. The most likely adverse effect is an allergy to soy protein.

Women and Minority Representation

The justification for this study is to evaluate an alternative to hormone replacement therapy in postmenopausal women. Thus, women will be studied exclusively. The minority distribution will reflect this community.

Population estimates by race and hispanic origin in Tippecanoe County 1996 estimates.

Population	*No*	*%*	*Target Recruitment %*
Total	*138324*	*100*	*100*
White	*128633*	*93*	*80*
Black	*3056*	*2.2*	*10*
Am. Ind. Eskimo, Aleut	*370*	*0.3*	*0*
Asian, Pac. Island	*6265*	*4.5*	*10*
Hispanic	*2840*	*2.1*	*0*
No Hispanic	*135400*	*97.9*	*100*

D) Procedures for Recruitment of Subjects

Subjects will be recruited through radio and newspaper announcements and campus mailings.

Advertisements for Research Subjects

Soybeans and Bone Health

Seeking volunteer subjects to participate in a study of the effect of soybean products on calcium nutrition for bone health. Subjects must be healthy, women at least 4 years past menopause not currently taking hormone replacement therapy. Subjects will be paid $750/year to complete testing of 3 products. If interested, contact:

Dr. Connie M. Weaver or _________, Project Director
Department of Foods and Nutrition
Purdue University, 1264 Stone Hall
West Lafayette, IN 47907-1264
765-494-0385

E) Procedure for Payment of Subjects

Subjects will be paid $150 for each 50 day baseline and treatment period completed and $50 for each 50 day recovery period completed. Thus, a subject can earn up to $750/year.

F) Confidentiality

In any publication, the identity of the subject will not be revealed. Subjects records will be stored in a locked file cabinet or locked room.

G) Potential Risks for Subjects.

Drawing blood poses a risk of bruising. Blood will be drawn by a trained

phlebotomist.

^{44}Ca to be administered orally in the absorption test is a stable isotope and poses no risk to subjects.

Subjects will be administered 100 nCi ^{41}Ca intravenously once two months prior to the start of the study. This represents a radioactive exposure of approximately 0.1 mRem. Additionally, 4.8 mRem will be received by bone densitometry of total body, spine and hip which is considered to be minimal risk. The iv will be prepared by sterile technique in a laminar flow hood and tested for pyrogens and sterility. The iv will be administered by a trained health care professional.

H) Benefits to be gained by the individual and/or society.

Society may gain knowledge that a natural constituent in food can produce some of the bone protecting effects now seen on estrogen replacement therapy.

Research subjects will learn about their general health.

I) Risks are minimal. Subject compensation is for exposure to slight radiation and the inconvenience of consuming soy products and providing urine samples and blood draws.

J) Procedures to obtain informed consent

Subjects who respond to the advertisement will be mailed or given an application to complete which includes the health screening instrument and diet records. Subjects who meet the criteria will be invited for a visit to Stone Hall for an orientation where they will be administered a food frequency questionnaire and where the study and consent form will be explained.

K) A copy of the attached informed consent will be sent to the subject.

Purdue University
COMMITTEE ON THE USE OF HUMAN RESEARCH SUBJECTS
Revision Of Protocol Form

Principal Investigator: Dr. Connie M. Weaver

Protocol Title: Effect of Soy Products on Calcium Metabolism in Humans

Protocol Reference No.: 0504001933 Phone/Fax 765-494-8231

Department/Building: Foods & Nutrition/ Stone Email: weavercm@purdue.edu

---

1. Please list any research personnel being added to the protocol **and** their role in the research project.

Key Personnel:
Cindy Nakatsu, Professor Agronomy, will supervise the microbial analysis described with this amendment (Agronomy, Lilly, 62997, 62926, cnakatsu@purdue.edu)
Annie Elble, Graduate Student, will help coordinate the study, interact with subjects, analyze specimens and data. (F&N, Stone, 40385, 40906, aelble@purdue.edu)
Doug Maish, EMT-P will act as phlebotomist and be in charge of distributing the tablets during each treatment phase. (F&N, Smith, 61877, 62257, maish@purdue.edu)

Non-Key Personnel:
LeeColeLegette, graduate student, will analyze microflora changes in feces collected during the equilibration phase. (F&N, Stone, 40385, 40906, llegette@purdue.edu)

3. Describe the rationale for the revision: While we know that different people metabolize certain phytoestrogens in soy differently and that this causes variation in the gut microflora we need to clarify the exact changes that occur to enable us to begin to characterize the mechanism that may result in the effects of these phytoestrogens on bone loss. Because one of the screening procedures in this study is to classify subjects into equol and non-equol producing groups this is an opportune time to identify specific changes in gut microflora.

4. Describe changes to the procedures:

PROCEDURES

1. During a previously described equilibration period we will ask the subjects to refrain from eating any soy products for one week. After this week, each subject will be asked to collect 7 fecal samples over a minimum of the next 7 days (no more than one sample per day). The subject will then be asked to consume one soy bar each day for three consecutive days. On the 4th day, she will collect my first urine void for analysis of soy metabolites. She will continue to consume one soy bar per day over at least the next 7 days or until she has collected 7 fecal samples (no more than one fecal sample per day) Appropriate containers will be provided to the subjects and the specimens will be picked up by the study staff according to a pre-arranged schedule.

2. Because this is a lengthy study we wish to repeat the bone density analysis at the end of the study to verify bone loss or gain.

COMPENSATION:
Subjects will receive $75 for the fecal collections and $75 for the baseline period. All other payment schedules will remain the same.

5. Describe changes to recruitment and targeted subject population:

6. Submit copies of all instruments and consent forms affected by this revision.
See attached.

Principal Investigators Signature: Connie Weaver ________________________________ Date: ______________
